# Evaluation and analysis of theoretical knowledge proficiency and practical skills among parasitic disease practitioners in Hainan province: a cross-sectional survey

**DOI:** 10.3389/fpubh.2025.1600908

**Published:** 2025-08-06

**Authors:** Wenjuan Liang, Guangda Xu, Xiaomin Huang, Shuming Yang, Rongguang Zhang, Yuchun Li

**Affiliations:** ^1^Department of Epidemiology, Public Health School, Hainan Medical University, Haikou, China; ^2^Hainan Provincial Center for Disease Control and Prevention, Haikou, China

**Keywords:** parasitic diseases, competence analysis, laboratory diagnosis, accuracy, Hainan province

## Abstract

**Objective:**

This study aimed to evaluate both theoretical knowledge proficiency and practical skills among practitioners specializing in parasitic diseases in Hainan province, providing essential groundwork for ongoing capacity enhancement efforts.

**Methods:**

A cross-sectional study using two-stage stratified random sampling was conducted in Hainan province. City or county-level representatives from Centers for Disease Control and Prevention and other institutes, as well as medical institutions focusing on parasitic diseases, participated in this subnational assessment process. They were evaluated across several key areas, including theoretical knowledge, smear sample examination, and microscopic identification, with a specific focus on *Plasmodium* species and parasite eggs.

**Result:**

A total of 54 county-level representatives participated in this study, with 23 from 41 CDCs and 31 from medical institutes. Significant differences were observed in theoretical knowledge scores across various parasitic species (*χ*^2^ = 81.563, *p* < 0.001), with Plasmodium spp. achieving the highest score at 82.22%, while other species scored the lowest at 53.31%. The average scores for malaria blood films and modified Kato–Katz smears were reported as 6.39 ± 2.35 and 9.13 ± 1.54, respectively. The radar chart revealed that blood film staining had the lowest scoring rate at 41.96%, while extruded film achieved the highest at 86.16%. Significant differences were also found among different *Plasmodium* species (*χ*^2^ = 35.161; *p* < 0.001), with *Plasmodium ovale* recording the lowest score of 0.00%. The eggs of roundworm had the highest positive detection rate at 82.61%, whereas the eggs of *Schistosoma japonicum* had the lowest at 5.89%. Furthermore, women outperformed men in total scores, smears, and microscopic examinations (*p* < 0.05), while results indicated higher performance in eastern regions compared to western ones (*p* = 0.039). According to their overall scores, 14.8% of participants achieved certification at Level 1, while 51.9% attained Level 2.

**Conclusion:**

These findings underscore an ongoing necessity to bolster both theoretical understanding and skill proficiencies related to parasitic diseases—particularly in malaria blood smear analysis, detection of *Plasmodium ovale*, and identification of *Schistosoma japonicum* eggs, especially among men and western Hainan’s practitioner community.

## Background

Parasitic infections remain a significant global health burden, particularly in developing nations, causing substantial morbidity and socioeconomic effects (1, 6). Despite extensive efforts such as nationwide vector control programs, precision surveillance systems, and cross-sector collaboration over several decades, China has not reported any indigenous malaria cases since 2017 and was officially certified malaria-free by the World Health Organization (WHO) on 30 June 2021 (3). However, the country continues to face challenges from various parasitic diseases, including not only imported malaria cases but also soil-transmitted helminthiasis and food-borne parasites (FBPs) (4), such as *Clonorchis sinensis, Schistosoma japonicum,* and *Necator americanus*.

Hainan Province, China’s southernmost tropical island, presents unique challenges related to parasitic diseases. Its tropical climate and environment are conducive to the breeding of *Anopheles dirus* and *Anopheles minimus*, while human behaviors, such as entering forested areas, further facilitate the transmission of malaria and other parasitic pathogens (5, 6). Historically, Hainan Island was endemic for *Plasmodium vivax* and *Plasmodium falciparum* (7). However, the geographical range of locally transmitted malaria has significantly contracted, with the last indigenous case of *Plasmodium malaria* reported in 2015, and no indigenous cases reported in 2016 (6). In 2018, the Chinese government approved the establishment of the China (Hainan) Pilot Free Trade Zone on Hainan Island, with a strategic focus on the development of international tourism and high-end medical services (8). The increase in international travel over the past decade has significantly contributed to the spread of parasitic diseases. Consequently, the increasing risks of imported parasitic diseases, such as *schistosomiasis*, *leishmaniasi*s, resurgence of controlled endemic diseases, and the emergence of novel pathogens, remain a critical concern, even in the post-elimination stage in Hainan Province. This evolving parasitic disease landscape has imposed greater demands and challenges on the region’s healthcare system to effectively manage imported cases from aboard (5).

Professional technicians constitute the primary workforce for addressing parasitic diseases. However, the competency level of these technicians, particularly in responding to parasitic diseases such as malaria, remains unclear in Hainan province. Therefore, our study aims to assess these critical capacities, including detection competency,‌ to inform targeted training programs and strengthen Hainan’s parasitic disease control framework.

## Methods

### Study population

**Figure 1:**
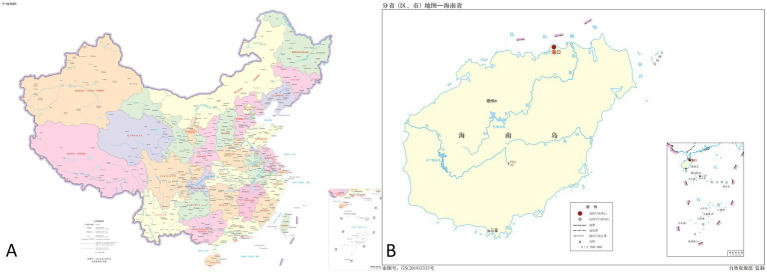
Maps in Hainan Island, China. **(A)** Map of China (GS(2022)4316); **(B)** Map of Hainan (GS(2019)3333): 海南岛 (Hainan island); 海口市 (Haikou city); 三亚市(Sanya city); 儋州市 (Danzhou city).

Provincial authorities delineated the scope and established the selection criteria: participants from city or county-level CDCs and institutes and medical institutions specializing in parasitic diseases were involved in this subnational evaluation process. Each city or county was tasked with selecting three representatives to partake in the assessment: one from the local Centers for Disease Control and Prevention (CDC), another from a local general hospital, and the third selected randomly. Then, participants were randomly selected from each city or county, and they subsequently underwent a standardized assessment in June 2022. For this cross-sectional study, demographic characteristics were extracted, including age, sex, education level, professional title, and institutional affiliation, along with location information. Participants were categorized into three geographical regions—east, west, and central—based on the location of their respective CDC or hospital, as shown in Figure 1.

### Study procedures

All participants were subjected to a competency assessment designed to evaluate their knowledge and skills in relation to the parasites, using a blind methodology.

### Theoretical knowledge assessment

The questionnaire development for the theoretical knowledge assessment followed a structured process. First, content domains were identified through expert consultation, covering parasite biology (life cycles and transmission routes), disease characteristics (etiology and clinical manifestations), diagnostic approaches (morphological identification and molecular methods), and treatment protocols and control measures. Second, the final assessment instrument consisted of 50 multiple-choice items (scored 0–100). Specifically, 26 questions addressed food-borne parasites, 5 focused on soil-borne parasites, 5 on *Plasmodium* species, 7 on other parasitic species, and 7 on multiple species combined. The electronic testing platform automatically scored responses and generated performance reports through the Laboratory Information Management System.

### Practical skills assessment

The skills assessment encompassed specimen preparation (malaria blood smear: 10 points and modified Kato–Katz smear preparation: 10 points) and microscopic identification (including microscopic examination of *Plasmodium*: 30 points and identification of key parasite eggs: 50 points), for a total score of 100. The skill assessment adhered to standardized criteria, which included uniform investigation methods, standardized equipment, and rigorous quality control.

All investigators completed a comprehensive training protocol followed by standardized competency assessments. Final proficiency ratings were determined using a hierarchical evaluation system: the composite score was computed through the sum of categorical scores, with subsequent tiered classification based on multidimensional performance criteria. Level 1 certification (advanced competency) required simultaneous achievement of ≥75th percentile thresholds in aggregate scores, domain-specific knowledge, and technical skill assessments. The intermediate proficiency designation (Level 2) was conferred when all evaluation metrics fell between the 50th and 75th percentiles. Complete quantitative thresholds and scoring matrices are detailed in Supplementary Table S1.

### Statistical analyses

Categorical variables were summarized as frequencies and percentages. Group comparisons were conducted using *χ*^2^ tests or Fisher’s exact tests, as appropriate. Continuous variables were presented as means with standard deviations and compared using the *t*-test and one-way ANOVA. Tukey’s multiple comparison test was applied for pairwise comparisons.

All the data were collected using Excel 2016, and statistical analyses were performed in SPSS22. A *p*-value of < 0.05 was considered statistically significant.

## Results

### Demographics characteristics

A total of 54 participants [15 male (27.8%) and 39 female (72.2%) individuals] were included in the full-process assessment of competency levels in knowledge and skills. Specifically, 23 participants (42.6%) were from the CDC, while 31 (57.4%) were from hospitals. Furthermore, 9 participants (16.7%) graduated from college departments, 44 (81.4%) from university departments, and only a small proportion (1.8%) obtained a master’s degree.

### Theoretical knowledge assessment

The total average score was 112.18 ± 36.45 points, with the theoretical knowledge component averaging 62.71 ± 21.53 points (Table 1). Further analysi*s* of scoring rates across different parasitic species showed a significant difference (*χ*^2^ = 81.563, *p* < 0.001) (shown in Supplementary Table S2). The highest scoring rate was recorded *for Plasmodium* spp. (82.22%), while the lowest was recorded for other species (53.31%).

**Table 1:** Competency results of 54 participants in Hainan province.

Variables	Total score (200 points)	Theoretical knowledge (100 points)	Blood smears		Microscopy	
			For malaria (10 points)	Kato–Katz smear (10 points)	Plasmodium (30 points)	Parasite eggs (50 points)
L1	14.8%	27.8%	42.6%	92.6%	20.3%	13.0%
L2	51.9%	42.5%	29.6%	5.5%	16.7%	27.8%
L3	31.5%	27.8%	22.2%	0%	16.7%	37.0%
L4	1.9%	1.9%	5.6%	1.9%	46.3%	22.2%

^*^: Mean ⦁ ( $\bar{x}$ ), standard deviation(SD).

### Practical skills assessment

#### Malaria blood smear and modified Kato–Katz smear

The mean scores for malaria blood smears and modified Kato–Katz smears were 6.39 ± 2.35 and 9.13 ± 1.54 points, respectively (Table 1). The radar chart indicated that the highest scoring rate for the malaria blood smear was observed in smear rules (97.39%), while the lowest was in blood film staining (41.96%), as shown in Supplementary Figure S1. In contrast, the highest scoring rate for the modified Kato–Katz smear was achieved in the placement of quantitative plates to fill fecal samples and manure scraping and sampling (100%), whereas the lowest was observed in extruded film (86.16%).

#### Microscopic examination of plasmodium and key parasite eggs

**Figure 2:**
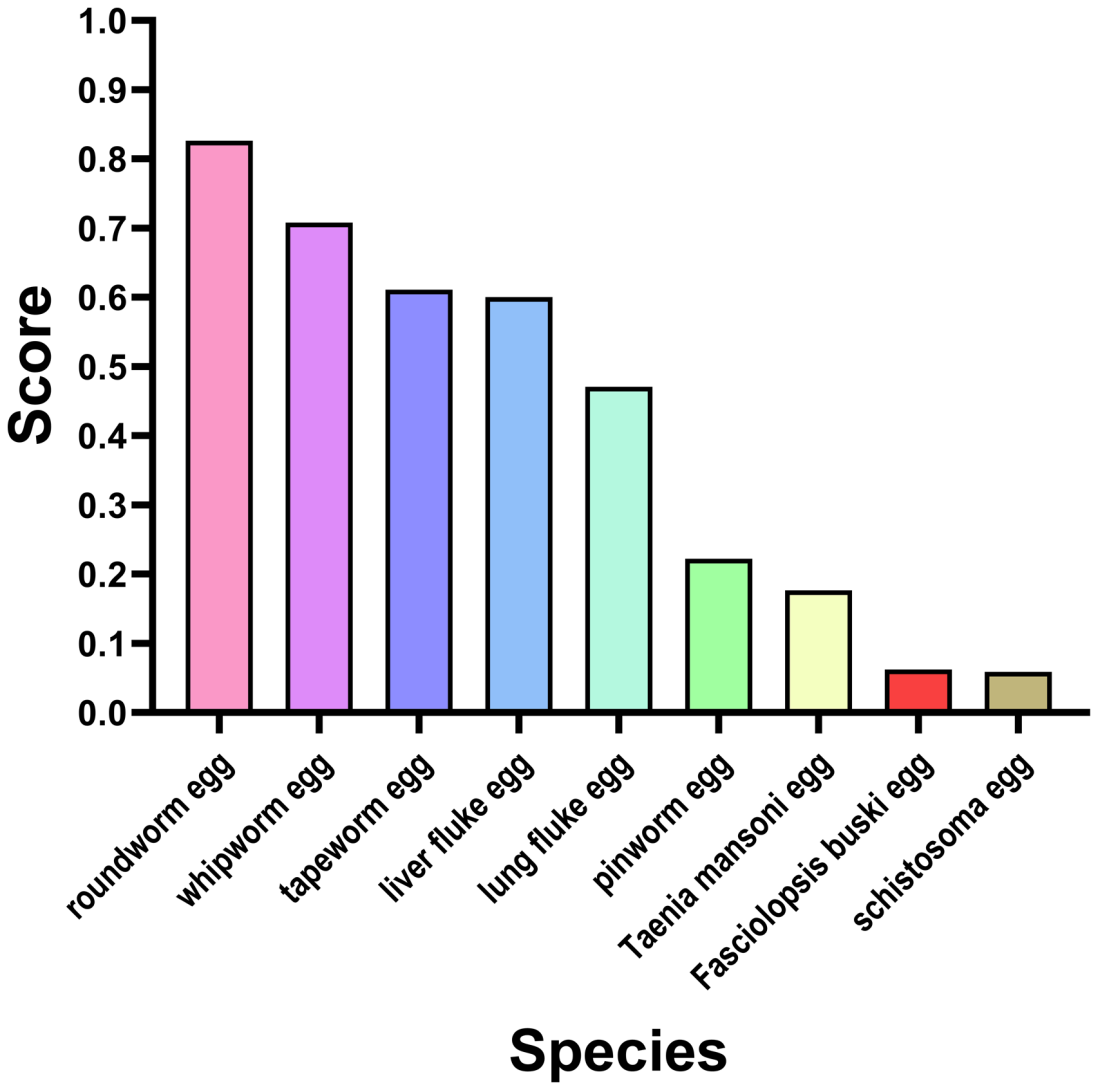
Scoring rate of egg detection of different priority parasites. ‌Y‌: Scoring rate, indicating the detection scoring rate of parasite eggs. ‌X‌: Parasite egg species ranked by scoring rate in descending order.

The mean scores for microscopic examination of *Plasmodium* and key parasite egg*s* were 11.67 ± 9.92 and 22.26 ± 11.37 points, respectively (Table 1). Further analysis revealed a significant difference in scoring rates among various *Plasmodium* species (*χ*^2^ = 35.161, *p* < 0.001), as shown in Supplementary Table S3. The highest scoring rate among different plasmodium species was observed for *Plasmodium vivax* (51.85%), while no instances were recorded for *Plasmodium ovale* (0.00%). Figure 2 shows that the ova of roundworm had the highest positive detection rate at 82.61%, whereas the ova of *Schistosoma* had the lowest rate at 5.89%.

### Analysis of the associated factors of total scores

The mean total scores for the female group (118.75 ± 35.63 vs. 95.09 ± 33.93; *p* = 0.031), specimen preparation (16.13 ± 2.51 vs. 13.92 ± 3.71; *p* = 0.014), and microscopic examination (37.59 ± 18.29 vs. 24.40 ± 13.34; *p* = 0.014) were significantly higher than those for the male group (Table 2). Similarly, significant differences were observed in total score (*p* = 0.013), specimen preparation (*p* = 0 0.008), and microscopic examination (*p* = 0.005) among geographical areas, with pairwise comparison revealing significant disparities between the eastern and western regions in total score, specimen preparation, and microscopic examination (Supplementary Figure S2). Moreover, the eastern region differed significantly from the central region in terms of total score and microscopic examination, while statistical significance was also found for theoretical knowledge among educational groups (*p* = 0.045). No statistically significant differences were identified for other parameters.

**Table 2:** Analysis of the associated factors of competency results.

Variables	*n*	Theoretical knowledge [ $\bar{x}$ ± s]	*P*	Blood smears [ $\bar{x}$ ± s]	*P*	Microscopy [ $\bar{x}$ ± s]	*P*	Total score [ $\bar{x}$ ± s]	*P*
Sex									
Male	15	56.77 ± 23.06	0.210	13.92 ± 3.71	0.014*	24.40 ± 13.34	0.014*	95.09 ± 33.93	0.031*
Female	39	65.03 ± 20.77		16.13 ± 2.51		37.59 ± 18.29		118.75 ± 35.63	
Age group									
≤25	11	62.18 ± 21.40	0.947	13.56 ± 3.55	0.055	32.00 ± 13.59	0.378	106.74 ± 27.93	0.595
26 ~ 30	24	63.60 ± 17.89		16.26 ± 2.26		35.21 ± 17.33		115.08 ± 32.40	
31 ~ 35	11	60.27 ± 30.09		15.06 ± 3.96		27.46 ± 20.68		102.79 ± 49.17	
≥36	8	65.63 ± 22.02		16.61 ± 1.55		41.63 ± 20.85		123.86 ± 40.91	
Professional title									
Junior	44	60.64 ± 21.44	0.318	15.25 ± 3.29	0.243	31.31 ± 17.24	0.074	107.20 ± 35.48	0.097
Intermediate	5	68.60 ± 14.67		16.00 ± 1.50		40.80 ± 17.63		125.40 ± 20.73	
Senior	5	75.00 ± 29.39		17.66 ± 0.96		49.40 ± 21.70		142.06 ± 51.41	
Educational level									
College degree or below	9	49.81 ± 22.11	0.045*	14.94 ± 2.67	0.621	28.75 ± 23.05	0.213	93.50 ± 43.23	0.056
Undergraduate degree	44	64.20 ± 20.41		15.57 ± 3.11		34.22 ± 16.76		113.99 ± 33.64	
Post-graduate degree or above	1	100.00 ± 0.00		18.00 ± 0.00		62.00 ± 0.00		180.00 ± 0.00	
Institution									
CDC	23	63.08 ± 20.35	0.916	14.86 ± 2.93	0.153	37.67 ± 18.27	0.173	115.61 ± 35.43	0.541
Hospital	31	62.45 ± 22.78		16.05 ± 3.04		30.93 ± 17.43		109.43 ± 37.62	
Regions									
Eastern	18	227.75 ± 39.78	0.073	51.65 ± 2.76	0.008*	140.50 ± 36.80	0.005*	419.90 ± 75.16	0.013*
Middle	18	163.75 ± 41.88		46.02 ± 4.55		91.33 ± 35.43		301.10 ± 69.15	
Western	18	173.08 ± 59.32		42.00 ± 5.89		73.50 ± 13.37		288.58 ± 75.33	

^*^P < 0.05 was determined to represent a statistically significant difference.

### Total competency analysis

The competency results of knowledge and skills are shown in Table 1. According to the total score, 14.8% of the participants were certified at Level 1, 51.9% at Level 2, and the other participants were certified at either Level 3 or Level 4.

## Discussion

Despite the exceptional improvements that have been accomplished in fighting parasites over the last 60 years in China, imported parasitic disease cases are inevitable, and such cases have increasingly been reported as a result of enhanced globalization and international or regional cooperation (9). The prompt and precise diagnosis of symptomatic and asymptomatic carriers is the key aspect in preventing the transmission of parasitic disease. Our study addressed this critical gap by systematically evaluating—for the first time in Hainan province—the diagnostic capabilities of frontline laboratory personnel, revealing both strengths and vulnerabilities in their parasite identification capacity.

The assessment used knowledge examination and practical operation training, including 54 representatives from the CDC and hospitals, to determine competency levels and strengthen the laboratory network for parasite diagnosis in Haikou. The average score for theoretical knowledge was 62.71 ± 21.53, suggesting that participants had a good grasp of the basic theory of parasitic diseases, consistent with findings from Jiangsu and Shandong provinces (10, 11). The sources of theoretical knowledge included food-borne parasites, soil-borne parasites, *Plasmodium,* and other species. Notably, the comparatively lower performance on “other species” (53.31%) likely reflected their lower epidemiological prevalence relative to more frequently encountered parasite categories. These findings underscore the need for targeted training programs focusing on rare parasitic species to strengthen comprehensive diagnostic capacity.

The specimen preparation results revealed divergent performance between techniques: modified Kato–Katz smear achieved moderately high scores (9.13 ± 1.54), whereas malaria blood smears demonstrated notably lower proficiency (6.39 ± 2.35). Particularly concerning was the blood film staining step (41.96% pass rate), falling below minimum competency thresholds. This deficiency warrants attention given the critical role of staining quality in the microscopic diagnosis of parasitic diseases (12). Interestingly, the modified Kato–Katz technique showed consistently strong performance across all evaluation metrics(9.13 ± 1.54), even surpassing reported benchmarks from Shandong province (8.92 ± 1.15) (10). This disparity likely reflects Hainan province’s established training protocols and routine clinical application of the Kato–Katz method for intestinal parasite screening.

Conventional smear and light microscopy are considered the “gold standard” for parasite identification and confirmation (13). However, the average scores for microscopic examination of *Plasmodium* and key parasite eggs were lower compared to those for smear preparation, especially for the different *Plasmodium* species and *Plasmodium ovale*. These findings demonstrate low competency of *Plasmodium* species and ova of *Schistosoma* identification using microscopes. However, there was an outbreak of *Plasmodium malariae* infection among forest goers in Sanya City of Hainan Island in 2015, and an innovative three-layer strategy targeted at forest goers was implemented, which was effective in blocking the outbreak by *P*. *malariae* among forest goers in Hainan in the malaria elimination stage (14). In China, only Sanya has reported locally sequentially indigenous cases of *P*. *malariae* (15). On the other hand, in recent years, the incidence of food-borne and soil-borne parasites has shown a downward trend (16). Thus, the identification of rare parasite species and eggs still remains a weak link in the prevention and control of parasites in our province. Therefore, for the identification of parasite eggs, it is essential to conduct a series of educational technology training to improve professional technicians’ ability to master microscopic technologies.

To determine the impact of different factors, a significance analysis was performed. Statistical analysis revealed significant gender-based disparities in assessment outcomes, with female participants demonstrating superior performance in total scores (*p* < 0.05), specimen preparation, and microscopic examination. These findings are consistent with previous studies by Cai (17), which reported in 2016 that theoretical and microscopic examination scores of female participants were significantly higher than those of male participants (*U* = 753.50, 775.50, *p* < 0.05), and in 2019 that female participants outperformed male participants in malaria slide-making (*U* = 775.50, *p* = 0.01). This convergent evidence suggests that women tend to be more attentive and patient in smear processing and microscopic techniques than men. Based on these robust findings, we recommend establishing peer-learning dyads that pair high-performing female participants with male participants requiring improvement in future training programs. Such evidence-based strategies could effectively bridge the competency gap, while capitalizing on demonstrated female expertise in cytological diagnostics.

The regional performance disparities revealed significant differences in technical competencies between eastern and western regions (total score, specimen preparation, and microscopic examination, all *p* < 0.05). These findings align with previous observations that malaria-endemic regions (particularly Class I/II counties) and Global Fund-supported provinces demonstrate superior slide preparation and microscopic reading skills (18). These findings underscore the necessity for: (a) standardized competency-based training programs in western regions and (b) equitable resource allocation to establish a sustainable parasitic disease surveillance network.

The total average score was 112.18 ± 36.4, with 14.8% of participants certified at Level 1, 51.9% at Level 2, and 30% scoring below proficiency thresholds—particularly alarming given Hainan’s status as China’s only tropical island with unique parasite transmission risks. The results revealed that the majority of participants were competent in theoretical knowledge and practical operations. However, 30% of participants had lower scores, indicating potential gaps in competency for theoretical knowledge and practical skills related to parasitic diseases. It is imperative to pay attention and make efforts to provide support to help these individuals develop into skilled professionals within their organizations.

There are still several shortcomings in the present study, which need to be further improved in the competency assessment of parasitic disease diagnosis in the future. First, the sample size was relatively small and insufficient to reflect the actual competency. Second, this study had a relatively large number of primary professional titles, but a small number of middle or senior professional titles. Finally, the assessment methodology could not capture the full spectrum of real-world diagnostic scenarios encountered in routine clinical practice.

## Conclusion

In conclusion, the findings suggest that there is still a need for sustained strengthening of theoretical knowledge and practical operation competency in parasitic diseases through training and practice, and regular quality assurance supported by adequate policy support, particularly in the areas of malaria blood smear analysis, detection of *Plasmodium ovale,* and identification *Schistosoma* ova, especially among practitioners in Western Hainan.

## Data Availability

The original contributions presented in the study are included in the article/Supplementary material, further inquiries can be directed to the corresponding author.
