# Supplementary material for: Evaluation and analysis of theoretical knowledge proficiency and practical skills among parasitic disease practitioners in Hainan province: a cross-sectional survey
Source: Front Public Health. 2025 Aug 6;13:1600908. doi: 10.3389/fpubh.2025.1600908 (PMC12364811; doi:10.3389/fpubh.2025.1600908)
Supplement: Supplementary file 1 [file Data_Sheet_1.pdf]

Supplement materials

Table S1 Competency levels and criteria.

Table S2 Comparison of the scoring rates of theoretical knowledge for different species among all participants.

Table S3 Comparison of scoring rates of different Plasmodium species in all participants.

Figure S1 Radar chart of scoring rate for each step of malaria blood and Kato-Katz smear.

Figure S2 The competency comparison of the different regions in Hainan Province.

Table S1 Competency levels and criteria.

Competency level	Total (200 scores)	Theoretical knowledge (100 scores)	Blood smears (20 scores)	Microscopy（80 scores）
L1	≥ 150	≥ 75	≥ 15	≥ 60
L2	100 to < 150	50 to < 75	10 to < 15	40 to < 60
L3	50 to < 100	25 to <50	5 to <10	20 to <40
L4	< 50	< 25	< 5	< 20

L1-L4 was categorized into four grades (L1=75-100%, L2=50-75%, L3=25-50%, L4=0-25%).

Table S2 Comparison of the scoring rates of theoretical knowledge for different species among all participants.

	scoring	no score	scoring rates	χ^2	*P*
geo-nematodes#	332	208	61.48%		
foodborne parasites*	917	757	54.78%	81.563	<0.001
plasmodium§	222	48	82.22%		
other species$^{\theta}$	403	353	53.31%		

# geo-nematodes include hookworms, roundworms, Trichuris trichiura, and Enterobius vermicularis;
* foodborne parasites include Schistosome, T.spiralis, Fasciolopsis buski, bladder worm, Tapeworm, Spirometra mansoni, Echinococcus granulosus, Clonorchis sinensis, Paragonimus westermani, Angiostrongylus cantonensis, and Toxoplasma gondii.;
§ plasmodium include Plasmodium falciparum, Plasmodium vivax, Plasmodium malariae, Plasmodium ovale;
θ other species involve Leishmania protozoa, filarial worms, and Hymenolepis diminuta, etc.

Table S3 Comparison of scoring rates of different Plasmodium species among all participants.

species	scoring	no score	scoring rates	χ^2	*P*
Plasmodium falciparum	45	48	48.39%		
Plasmodium vivax	28	26	51.85%		
Plasmodium malariae	5	15	25.00%	35.161	＜0.001
Plasmodium ovale	0	38	00.00%		
Negative specimen	21	47	30.88%		

a

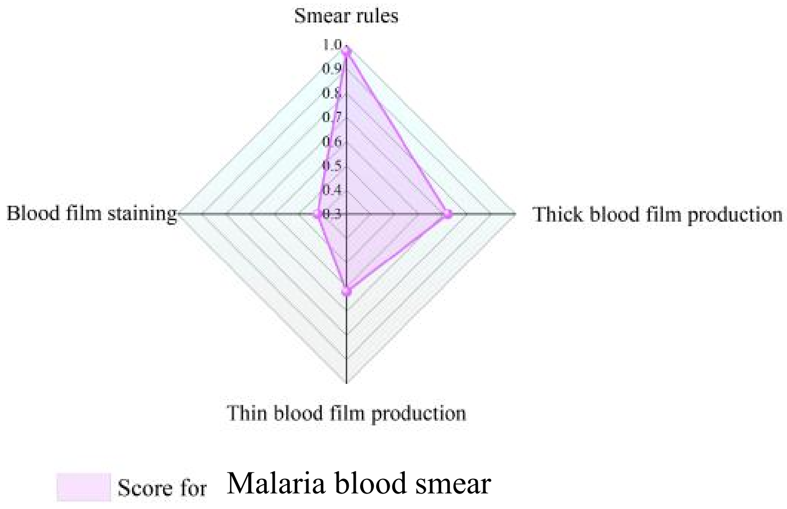

b

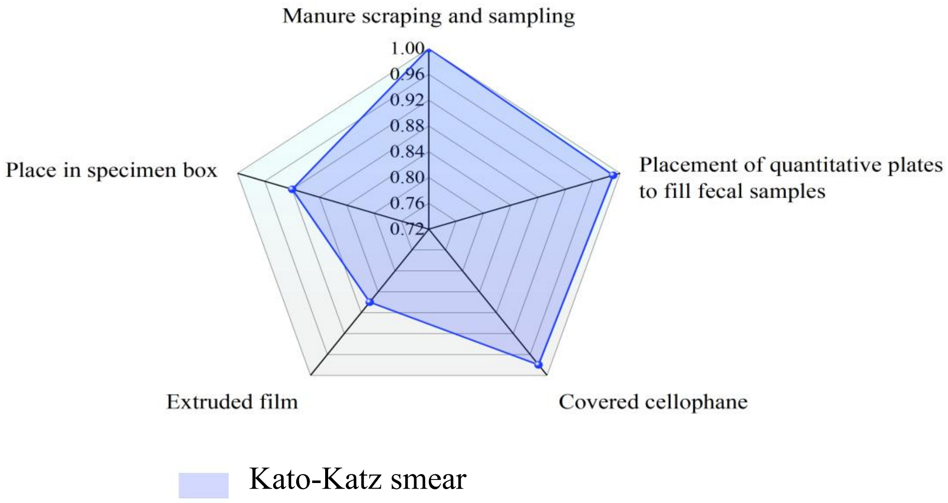

Figure S1 Radar chart of scoring rate for each step of malaria blood and Kato-Katz smear.

a. malaria blood smear; b. Kato-Katz smear

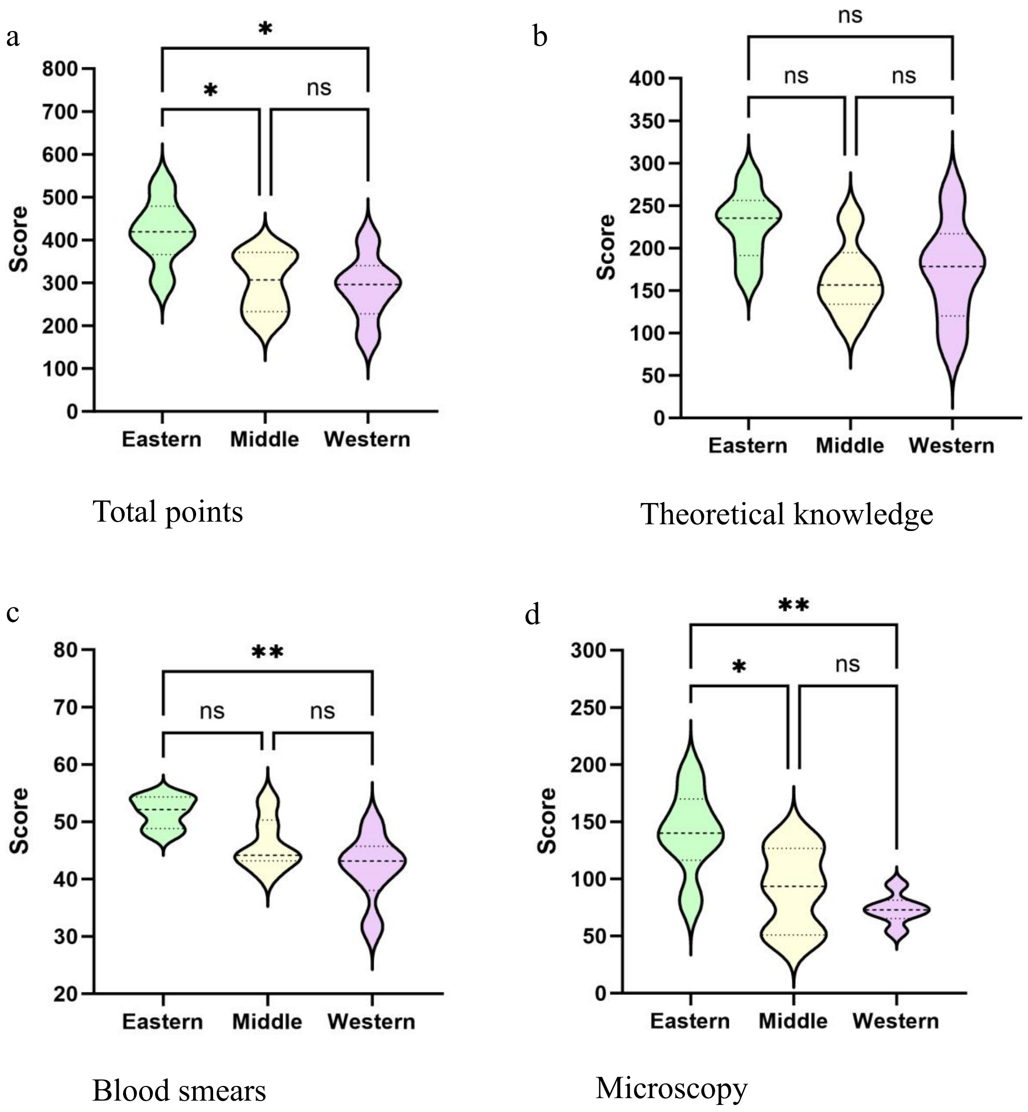

Figure S2 The competency comparison of the different regions in Hainan Province.

a. Total score; b. Theoretical knowledge; c. Blood smears; d. Microscopy
